# Redefining global health priorities: Improving cancer care in developing settings

**DOI:** 10.7189/jogh.04.010304

**Published:** 2014-06

**Authors:** Asad Moten, Daniel Schafer, Mauro Ferrari

**Affiliations:** 1 Institute for Translational Medicine and Novel Therapeutics, Healthnovations International, Houston, TX, USA; 2 Department of Primary Care Health Sciences, Nuffield Department of Clinical Medicine, University of Oxford, Oxford, UK; 3 National Cancer Institute Alliance for Nanotechnology in Cancer, National Institutes of Health, Bethesda, MD, USA; 4 Department of Nanomedicine, Houston Methodist Research Institute, Alliance for NanoHealth, Houston, TX, USA

Every year, more than 10 million people are diagnosed with cancer. Over half of them live in the developing world, where the cancer incidence rate has reached pandemic proportions. In 2008 there were upward of 12.4 million estimated new cases of cancer, with approximately two–thirds of the estimated 7 .6 million cancer deaths occurring in low–and middle–income countries [1]. Although low– and middle–income countries bear the majority of the disease burden, their health systems are inadequately prepared to address the challenge of care. Since cancer kills more people worldwide than HIV/AIDS, tuberculosis, and malaria combined, it needs to be a global health priority.

Low– and middle–income countries bear the greatest burden of new cancer cases as well as deaths, amid an ever–increasing total number of affected individuals worldwide. In 2012 an estimated 14 million new cases were reported, with this figure anticipated to rise to 22 million over the next twenty years [1]. Significantly, more than 60% of the world’s total cancer cases occur in Africa, Asia, Central America, and South America, while greater than 70% of the world’s cancer deaths come from these same regions [2]. By 2030 the developing world is expected to account for 70% of newly reported cancer cases. What is more, survival rates are meager in poor countries and on the rise in wealthy ones [3]. For a wide range of cancers, an individual’s likelihood of surviving is closely and positively related to country income (**Figure 1**** and ****2**).

**Figure 1:**
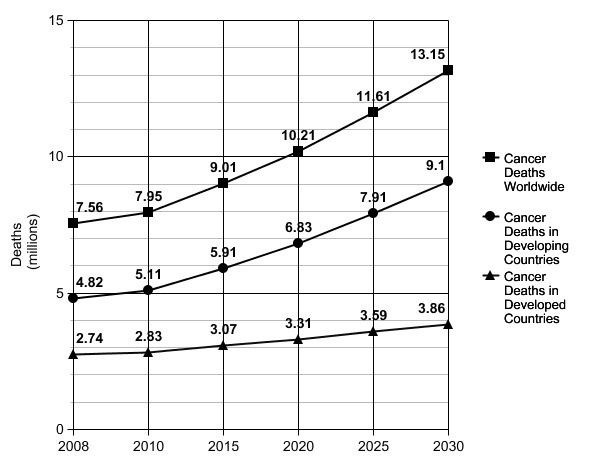
Countries in the developing world bear the greatest burden of new cancer cases as well as deaths. By 2030, the developing world is expected to account for 70% of newly reported cancer cases [3].

**Figure 2:**
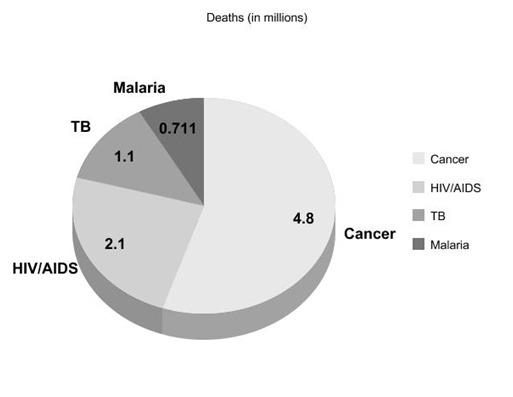
Deaths due to cancer, HIV/AIDS, tuberculosis (TB), and malaria in the developing world. Cancer kills more people worldwide than HIV/AIDS, TB, and malaria combined [3].

Part of the reason why low– and middle–income countries lag behind wealthy nations in cancer survival rates is a lack of awareness, prevention, and early detection, as well as inaccessibility of new and more effective treatments that have been developed in recent decades and made available to many in the first world. Greater than 30% of cancer–related deaths in the developing world are estimated to be preventable [2]. Thus, in the case of low– and middle–income countries, the demand for cancer prevention and treatment services is far from being met. With burgeoning survival rates among those suffering from AIDS and other diseases in low– and middle–income countries the prevalence of cancer will only increase, thereby resulting in an even greater need for cancer services in these areas.

While significant attention and financial support has resulted in expanded access to treatment for AIDS and other infectious diseases over the past decade, cancer is a concern that remains relegated to the periphery of the global health community. Despite its prevalence throughout the developing world cancer is conspicuously absent from central global health programs, including the United Nations Millennium Development Goals [4].

Cancer is a leading cause of mortality in low– and middle–income countries, equaling over five million annually. Disproportionate access to prevention and treatment, in addition to an ageing and growing population and decreasing mortality rates resulting from infectious diseases, will cause new cancer cases and cancer mortality to continue to rise in coming years. The global health community must take decisive action to bridge the cancer divide between wealthy and poor nations. Such action should take the form of increased access to drugs for treatment and palliation, expanded coverage for preventive and diagnostic services, as well as development and implementation of innovative health care delivery options to support rapid scale–up, while integrating access to patients in resource–poor regions through national health insurance systems.

## STRATEGIC APPROACH FOR CANCER CARE IN DEVELOPING SETTINGS

Provision of widespread and comprehensive cancer care is subject to criticisms that resemble those aimed at similar calls for control of HIV and MDR tuberculosis in previous years. In the case of HIV, some argued that antiretroviral treatment was too expensive to administer widely in the developing world, and that prevention, palliation, and less expensive treatments were the only justifiable and feasible options available [5]. In the cases of HIV and MDR tuberculosis, initially–expensive treatment became widely available when innovative treatment models and new investments were developed and implemented [6]. As a result of these efforts the number of HIV infected individuals receiving antiretroviral treatment increased more than 10-fold from 2003 to 2008, numbering, at the time, over 4 million [7].

Improving access to antiretroviral treatments for HIV patients models the fact that effective treatment for widespread diseases can be administered in low– and middle–income countries. However, as with AIDS, prevention is as necessary as treatment. Neglecting prevention results in unaffordable and overwhelming treatment demands just as neglect of care results in unnecessary death and suffering. An approach that integrates prevention and treatment is necessary in any effort to control cancer in the developing world.

**Figure:**
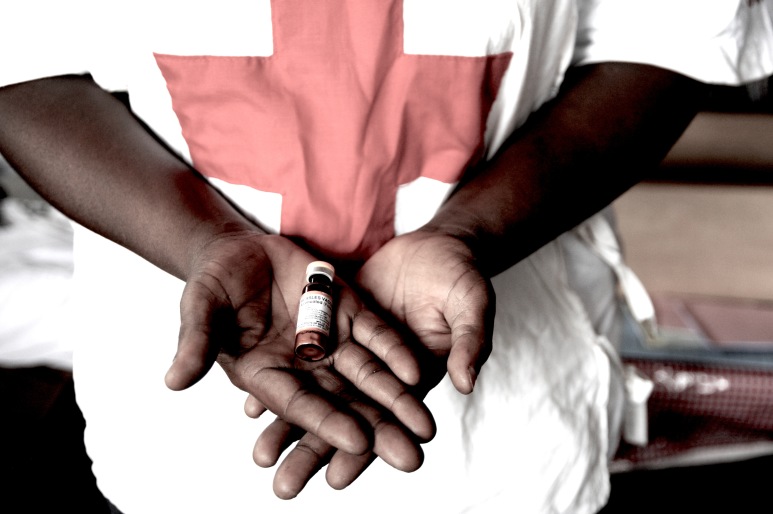
Photo: American Red Cross (http://media.redcross.org/sites/)

## PROMISING INTERVENTIONS FOR CANCER CARE IN RESOURCE–POOR SETTINGS

While direct access to oncological surgery facilities will remain a hurdle in low– and middle–income countries, existing initiatives have shown that health systems can be implemented in a cost–effective manner [8,9]. The key to these systems’ success lies in establishing functional primary care health infrastructures, particularly for cancers that are responsive to prevention and early detection efforts, as well as cost–effective generic drugs. Opportunities to educate, diagnose, and provide surgery, therapeutics, and palliative care to cancer patients in low– and middle–income countries should be identified and exploited. Moreover, an increase in the number of community health professionals and facilities, as well as access to prevention and early detection, will represent substantial progress in the local health infrastructure which will ultimately translate into improved health outcomes. While a robust health infrastructure has a direct impact on health outcomes, it also works to alleviate economic hardship through job creation in local economies resulting from technology transfer, an emergent service industry based on mobilization and utilization of health care professionals, and a host of other task–based positions that will emerge as a direct result of a functioning infrastructure [10]. Such an infrastructure, therefore, will serve to not only decrease mortality and morbidity due to the disease itself, but also effectively lessen the poverty–related hardship that can lead to disease faced, in particular, by marginalized communities.

Along with building the local health infrastructure, the potential of novel therapeutics needs to be exploited in developing settings. Nanotechnology is a multidisciplinary field, which includes a vast and diverse array of therapeutics derived from chemistry, physics, engineering, and biomedicine. Nanotechnology involves the manipulation of properties and structures at the nanoscale. Nanotechnology holds the promise of providing many significant benefits in medicine. For instance, Nanotechnology is being used for applications ranging from more effect drug delivery systems to highly sensitive pathogen detection platforms. To that end, the US National Institutes of Health has established the National Cancer Institute Alliance for Nanotechnology in Cancer in order to dedicate concerted efforts and resources for the development of cancer diagnostics and therapeutics. Such applications of nanotechnology in the diagnosis and treatment of disease are collectively known as “nanomedicine” [11].

Conventional medicine is largely inaccessible to patients in the developing world because of its dependence on technology, laboratory facilities, and/or highly–trained medical professionals. Conversely, nanomedicine, based predominantly on prevention and early management of disease, bypasses the need for scarcely–available, expensive laboratory equipment and medical expertise. Additionally, nanotherapeutics are fabricated in a highly–specific manner at a nanoscale level using bottom–up molecular manufacturing methods, which makes these nanomedicine drugs easier to manufacture, distribute, and subsequently administer to patients, all of which translate into improved health outcomes in resource–limited settings [12].

Currently, nanotechnology–based medicine is considered not only a significantly promising path to accurate cancer diagnosis and effective treatment, but also a plausible way to prevent and manage several chronic diseases. As nanomedicine evolves and becomes increasingly mainstream in clinical care, it is imperative to recognize its promise for combating the disproportionately high disease burden in the developing world. Applications of nanomedicine such as point–of–care diagnosis and effective nanoparticle–based drugs and vaccinations have the potential to revolutionize global health.

## SCALING–UP CANCER CARE IN THE DEVELOPING WORLD

**Figure 3:**
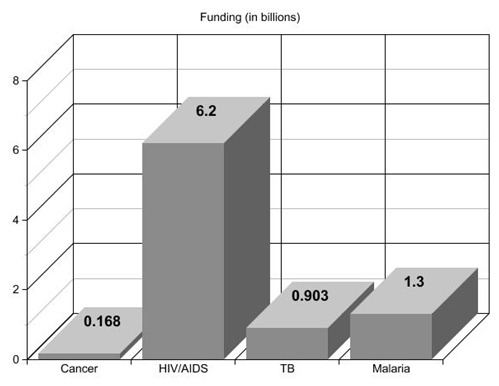
Funding (in US$) allocated to combat cancer, HIV/AIDS, tuberculosis (TB), and malaria in the developing world. Funding for prevention and treatment of cancer amounts to approximately 2% of what is put towards other diseases [13].

We know that cancer is among the most pressing, and growing, global health challenges [13]. However, as the need for access to cancer care in low– and middle–income countries increases, the amount of resources devoted to the developing world remains stagnant. Indeed, a mere 5% of all resources devoted to cancer care globally are allotted to the developing world [14]. Considering the continually–increasing number of individuals worldwide who will be diagnosed with cancer, lack of funding for care (**Figure 3**) coupled with limited access to oncology physicians and treatment centers in low– and middle–income countries forecasts a global health disaster–in–waiting.

Many who have been diagnosed with cancer live and die in rural, resource–poor settings. Given the rising number of cancer cases worldwide, and the knowledge that a wide range of cost–effective measures exist to meet the global challenge of cancer, rapid and comprehensive scale–up of education, prevention, treatment, and palliation should be undertaken immediately. By implementing education, prevention, and affordable treatment now, while simultaneously taking expeditious steps toward developing innovative funding strategies for currently cost–restrictive care, global availability of cancer care can become a reality unbounded by income level or location.

## CONCLUSIONS

In light of evidence that cancer prevention, diagnosis, treatment, and palliation is feasible across low– and middle–income countries, we call for the global health community to renew its efforts to make cancer drugs and other treatments affordable and accessible to all. We propose that cancer care and control be made available worldwide without delay, with particular attention paid to treating cancers that can be prevented or cured, and palliating those that cannot.

We propose immediate implementation of the following: First, define and create new infrastructure, train health professionals and paraprofessionals, and harness technology – especially telecommunications – to overcome geographic constraints, while identifying strategies that effectively alleviate the burden of cancer and expand health services in developing countries. Second, design and implement regional and global pricing and procurement standards to offer collective negotiation opportunities with an aim to reduce prices for essential services, drugs, and vaccines. Third, identify and implement innovative financing mechanisms to expand financial resources available for prevention, treatment, and palliation of cancer in low– and middle–income countries. Fourth, develop methods to support the creation of national policies surrounding cancer treatment to facilitate the implementation of the actions listed above.

Recalibrating global cancer care and control efforts could be transformative, but this kind of change will require a steadfast commitment to building integrated health infrastructures in the most marginalized settings, developing novel therapeutics specifically for resource–limited settings, and facilitating coordination between international organizations, bilateral donors, national research funding bodies, nongovernmental agencies, governments, and local regional, and global civil society organizations in order to secure funding, maximize efficiency of implementation, and ultimately improve health outcomes. Extension of cancer prevention, diagnosis, and treatment to those suffering or at risk is an urgent global health concern. Bold research, financing, and implementation strategies are essential to fill the cancer care and control void between low–, middle–, and high–income countries, and should be undertaken in earnest without delay.
