# **Moten A, Schafer D, Ferrari M. Redefining global health priorities: Improving cancer care in developing settings. *J Glob Health* 2014;4:010304corr1.**

**DOI:** 10.7189/jogh.04.010304corr1

**Published:** 2014-06

The authors are issuing a correction for the unintended oversight in attribution to this work [1]. To that end, the authors, with apologies, remove Paul Farmer and Jim Kim from the authorship byline and instead acknowledge that the paper was conceptualized in Health, Culture and Community: Case Studies in Global Health at Harvard University with the contribution and mentorship of Paul Farmer and Jim Kim. In their correction, the authors also include a citation from the Lancet [2] to their references and attribute photo credit to American Red Cross. The publisher has since corrected the online version of the article.
